# Single-cell transcriptional analysis of irradiated skin reveals changes in fibroblast subpopulations and variability in caveolin expression

**DOI:** 10.1186/s13014-024-02472-z

**Published:** 2024-06-26

**Authors:** Lionel E. Kameni, Michelle Griffin, Charlotte E. Berry, Siavash Shariatzadeh, Mauricio A. Downer, Caleb Valencia, Alexander Z. Fazilat, Rahim Nazerali, Arash Momeni, Michael Januszyk, Michael T. Longaker, Derrick C. Wan

**Affiliations:** 1 Hagey Laboratory for Pediatric Regenerative Medicine, Stanford University School of Medicine, Stanford, CA USA; 2 Geisel School of Medicine at Dartmouth, Hanover, NH USA; 3 Institute for Stem Cell Biology and Regenerative Medicine, Stanford University, Stanford, CA USA; 4 Division of Plastic and Reconstructive Surgery, Stanford University School of Medicine, 257 Campus Drive, GK 102, Stanford, CA 94305-5148 USA

**Keywords:** Radiation-induced fibrosis, Radiation therapy, Single-cell RNA sequencing, Caveolin

## Abstract

**Background:**

Radiation-induced fibrosis (RIF) is an important late complication of radiation therapy, and the resulting damaging effects of RIF can significantly impact reconstructive outcomes. There is currently a paucity of effective treatment options available, likely due to the continuing knowledge gap surrounding the cellular mechanisms involved. In this study, detailed analyses of irradiated and non-irradiated human skin samples were performed incorporating histological and single-cell transcriptional analysis to identify novel features guiding development of skin fibrosis following radiation injury.

**Methods:**

Paired irradiated and contralateral non-irradiated skin samples were obtained from six female patients undergoing post-oncologic breast reconstruction. Skin samples underwent histological evaluation, immunohistochemistry, and biomechanical testing. Single-cell RNA sequencing was performed using the 10X single cell platform. Cells were separated into clusters using Seurat in R. The SingleR classifier was applied to ascribe cell type identities to each cluster. Differentially expressed genes characteristic to each cluster were then determined using non-parametric testing.

**Results:**

Comparing irradiated and non-irradiated skin, epidermal atrophy, dermal thickening, and evidence of thick, disorganized collagen deposition within the extracellular matrix of irradiated skin were readily appreciated on histology. These histologic features were associated with stiffness that was higher in irradiated skin. Single-cell RNA sequencing revealed six predominant cell types. Focusing on fibroblasts/stromal lineage cells, five distinct transcriptional clusters (Clusters 0–4) were identified. Interestingly, while all clusters were noted to express Cav1, Cluster 2 was the only one to also express Cav2. Immunohistochemistry demonstrated increased expression of Cav2 in irradiated skin, whereas Cav1 was more readily identified in non-irradiated skin, suggesting Cav1 and Cav2 may act antagonistically to modulate fibrotic cellular responses.

**Conclusion:**

In response to radiation therapy, specific changes to fibroblast subpopulations and enhanced Cav2 expression may contribute to fibrosis. Altogether, this study introduces a novel pathway of caveolin involvement which may contribute to fibrotic development following radiation injury.

**Supplementary Information:**

The online version contains supplementary material available at 10.1186/s13014-024-02472-z.

## Introduction

Over recent decades, survival rates of several cancer types have continued to improve, with a shift toward an increasingly tailored use of radiotherapy playing a significant role in enhanced outcomes. Radiation therapy alone, or often combined with other treatment modalities such as precision immunotherapy and chemotherapy, has revolutionized cancer treatment, leading to unprecedented long-term survival benefits for cancers of the head and neck, prostate, lung, and breast [1–3]. Despite major advances made in radiotherapy techniques, a substantial proportion of patients nonetheless experience deleterious effects. Collateral skin and soft tissue injury is often noted in both acute and chronic phases of treatment. Skin changes after irradiation may include epidermal thinning, dermal thickening, atrophy of hair follicles/sebaceous glands, hair loss, increased collagen density, and decreased vascularization [4]. Over time, skin injury may also progress to pathological radiation-induced fibrosis (RIF).

While some of the basic concepts of RIF are known and well-documented in the literature, it is increasingly understood that RIF is a multifactorial process, and accumulating evidence suggests that fibroblasts are heavily implicated [5, 6]. Furthermore, anatomically distinct subsets of fibroblasts have been found to promote scarring following excisional skin wounding, and transcriptional analyses of these wounds have described functionally distinct subpopulations that may differentially contribute to scar formation [7, 8]. Though similar studies in radiation-injured skin is limited, as highlighted in a study by Straub et al., fibroblasts are responsible for a number of functions that contribute to the pathogenesis of RIF [9]. More specifically, in response to transforming growth factor beta, fibroblasts exhibit a phenotypic change into protomyofibroblasts and eventually mature into myofibroblasts [9, 10]. Myofibroblasts in turn secrete excessive amount of highly disorganized collagen framework as well as other extracellular matrix components. This is coumpounded by a reduction of remodeling enzymes such that there is net gain of extracellular matrix (ECM) [11, 12]. The culmination of these negative effects of radiation manifests as increased tissue stiffness and thickness characteristic of chronic RIF, which may result in cosmetic and functional impairment, with joint contracture and limited range of motion, that significantly impacts quality of life. In addition, RIF may present a major surgical challenge for patients needing post-oncologic reconstruction. This is thought to be because radiated tissues have decreased microvascular perfusion, contributing to complex soft tissue defects with higher rates of wound healing complications [11]. 

There is currently a paucity of effective treatment options that can reduce or reverse radiation-induced skin fibrosis [13], likely due to the continuing knowledge gap surrounding the cellular mechanisms involved. As such, in the present study, detailed analyses of irradiated and non-irradiated human skin samples were performed incorporating histological and single-cell transcriptional analysis to identify novel features guiding development of skin fibrosis following radiation injury. By providing deeper insights into the pathobiology of RIF, more effective therapeutics may thus be developed.

## Materials and methods

### Patient samples

Paired irradiated and contralateral non-irradiated skin samples were obtained from from female patients (*n* = 6, mean age = 53 ± 8.6 years of age) following unilateral mastectomy and radiation therapy undergoing subsequent post-oncologic autologous bilateral breast reconstruction, at which time skin samples were obtained (Table 1). All samples were each divided for histologic staining, mechanical testing, and transcriptional analysis. Handling of specimens was in accordance with the Stanford University Administrative Panel on Human Subjects Research Insitutional Review Board approved protocols.

**Table 1:** Patient demographics

Patient Number	Age	Gender	Dose (cGy)	Fractions	XRT Laterality	Post-XRT Time (months)
1	53	F	4000	16	L	18
2	43	F	5000	25	R	4
3	47	F	5000	25	R	6
4	68	F	5100	28	R	7
5	56	F	5000	25	L	12
6	51	F	4800	24	R	9

### Tissue processing and sectioning

Tissue samples were fixed in 10% neutral buffered formalin (Cat#5701; Thermofischer Scientific, Waltham, MA) at 4˚C for a minimum of 18 h, washed with PBS, dehydrated in gradients of alcohol using an automated Spin Tissue Processor (ThermoFisher Scientific, Waltham, MA), and embedded in paraffin blocks. Paraffin blocks were cut into 8 μm sections, placed in a warm water bath at 40 °C, and mounted onto Superfrost™ Plus adhesive slides which were then baked at 40 °C overnight.

### Histological analysis

Paraffin sections of skin specimens were stained with Hematoxylin and Eosin (H&E), Masson’s Trichrome (TC), and Picrosirius Red (Picro) to assess for dermal thickness, collagen density, and differences in ECM collagen features, respectively. The dermis was defined as the vertical distance from the basal layer of the epidermis to the underlying hypodermis. Collagen density of TC stained images was calculated from the density of blue in each image as assessed by ImageJ (US NIH, Bethesda, MD) color Deconvolution plugin. For Masson’s Trichrome and H&E stains, slides were imaged using a Leica DMI4000 B inverted microscope (Leica Microsystems, Wetzlar, Germany) with the 10X objective. Sections (*n* = 5) from each skin sample were randomly selected from the entire specimen for histologic analyses, yielding a total of 30 irradiated and 30 non-irradiated sections quantified. Picrosirius red-stained slides were captured (100 images per condition) at 40X using a polarizing filter. These polarized images were subsequently color deconvoluted, binarized, and skeletonized in MATLAB (Mathworks, Natick, MA) through a machine learning algorithm [14]. From the skeletonized images, 294 features (width, length, brightness, orientation, diameter, among others) of green and red collagen fibers were extracted, measured, and reduced by Uniform Manifold Approximation and Projection (UMAP) [15]. In the process, two dimensional plots are generated for visualization of collective differences in the collagen fiber network patterns between groups [16, 17]. 

### Evaluation of the mechanical properties of the skin

Skin samples were kept in Dulbecco’s Modified Eagle’s Medium solution on ice until tested post-harvest using the MTS Bionix 200 mechanical testing system (MTS Systems Corporation, Eden Prairie, MN) [18]. The underlying muscle was removed in order to measure skin properties only. The ends of each sample were clamped to a tensile tester and each specimen was stretched at a rate of 0.2 mm/s until failure using a ± 10 N load cell. Young’s Modulus was calculated using MATLAB, based on measured width, length, and thickness of each sample generated from the MTS TestWorks software platform.

### Immunohistochemistry

Three sections from each skin sample randomly selected from the specimen were deparaffinized with xylene, gradually rehydrated with decreasing concentrations of ethanol, and then treated with Trypsin Antigen-Retrieval Solution (No. Ab970; Abcam). Sections were blocked with 1X Power Block (No. HK083-50 K; BioGenex) and incubated for 1 h at 37 °C with unconjugated primary antibody anti-CD31 (No. Ab28364; Abcam), anti-calveolin-1 (No. Ab17052; Abcam), or anti-caveolin-2 (No. Ab97476; Abcam) diluted at 1:100 in 0.1X Power Block. After washing in phosphate-buffered saline (No. 10,010,023; Gibco), the tissue sections were incubated for 1 h at 37 °C with an anti-rabbit secondary antibody Alexa Fluor 594 (Cat#A11037; ThermoFisher Scientific) or anti-mouse secondary antibody Alexa Fluor 647 (No. Ab150115; Abcam) diluted at 1:200 in 0.1X Power Block. Specimens were once again washed in phosphate-buffered saline and then counterstained with DAPI Fluoromount-G. Images were taken with an inverted confocal microscope (LSM 880; Leica Microsystems) using standard field size (1,024 × 1,024) for all images.

### Single-cell RNA data processing, quality control, dimension-reduction, and clustering

Tissue from all 6 paired irradiated and non-irradiated specimens were mechanically digested with scissors followed by Collagenase II (Cat No. 17,101,015; ThermoFisher Scientific) and IV (Cat No. 17,104,019; ThermoFisher Scientific) in DMEM (GIBCO; Fisher Scientific). Samples were added to an orbital shaker at 150 rpm for 90 min at 37^o^C, resuspended in FACS buffer, and filtered through 70 μm and then 40 μm cell strainers. Cell counts were performed to determine adequate cell survival.

Single-cell RNA-seq (scRNA-seq) and associated quality control were performed on unsorted cells at the Stanford Functional Genomics Facility using the 10x Chromium Single Cell platform (Single Cell 3′ v3, 10X Genomics, Pleasanton, CA). After scRNA-seq, base calls were converted to reads using the Cell Ranger (10X Genomics; version 3.1) implementation of mkfastq and then aligned against the Cell Ranger GRCH38 human genome, using Cell Ranger’s count function with SC3Pv3 chemistry and a minimum of 5,000 expected cells per sample [14]. Thereafter, cells were filtered by unique molecular identifiers counts and the mitochondrial content ratio. Only cells with the mitochondrial content below or equal to 10% were retained. Filtering was followed by dimension-reduction and clustering using Seurat v3.1.2. Specifically, the ‘NormalizeData’ and ‘ScaleData’ functions were applied for the normalization and scaling of all gene expression. Next, we performed PCA analysis such that the top 15 principal components were selected to separate cells into clusters using Seurat’s ‘FindClusters’ function and visualized in a two-dimensional space, using a Louvain implementation of Seurat’s UMAP algorithm. SingleR was then applied using the Human Primary Cell Atlas reference dataset to ascribe cell type identities to each cluster.

We then focused specifically on fibroblasts, which were isolated *in silico* using the cell type identities described above. Fibroblast data from both IR and NIR tissue were separately normalized and re-clustered, as described above.

### Identification of differentially expressed genes

Differentially expressed genes (DEGs) were defined as genes with expression in more than 10% of the cells in a cluster and with a log fold change threshold of 0.25. Seurat’s native ‘FindMarkers’ function screened genes based on the receiver operating characteristic test with default parameters to identify DEGs for each cluster.

### Statistical analysis

Statistical analysis was done with GraphPad Prism v9.5.1 (GraphPad, Boston, MA). When applicable, data were expressed as mean and standard error of the mean. Normality was evaluated using a Shapiro-Wilk test and means were compared using a paired two-tailed student’s t-test. A value of *p < 0.05 was considered significant. Details are specified in each figure’s caption.

## Results

### Radiation-induced changes in dermal architecture and skin biomechanical analysis

**Fig. 1:**
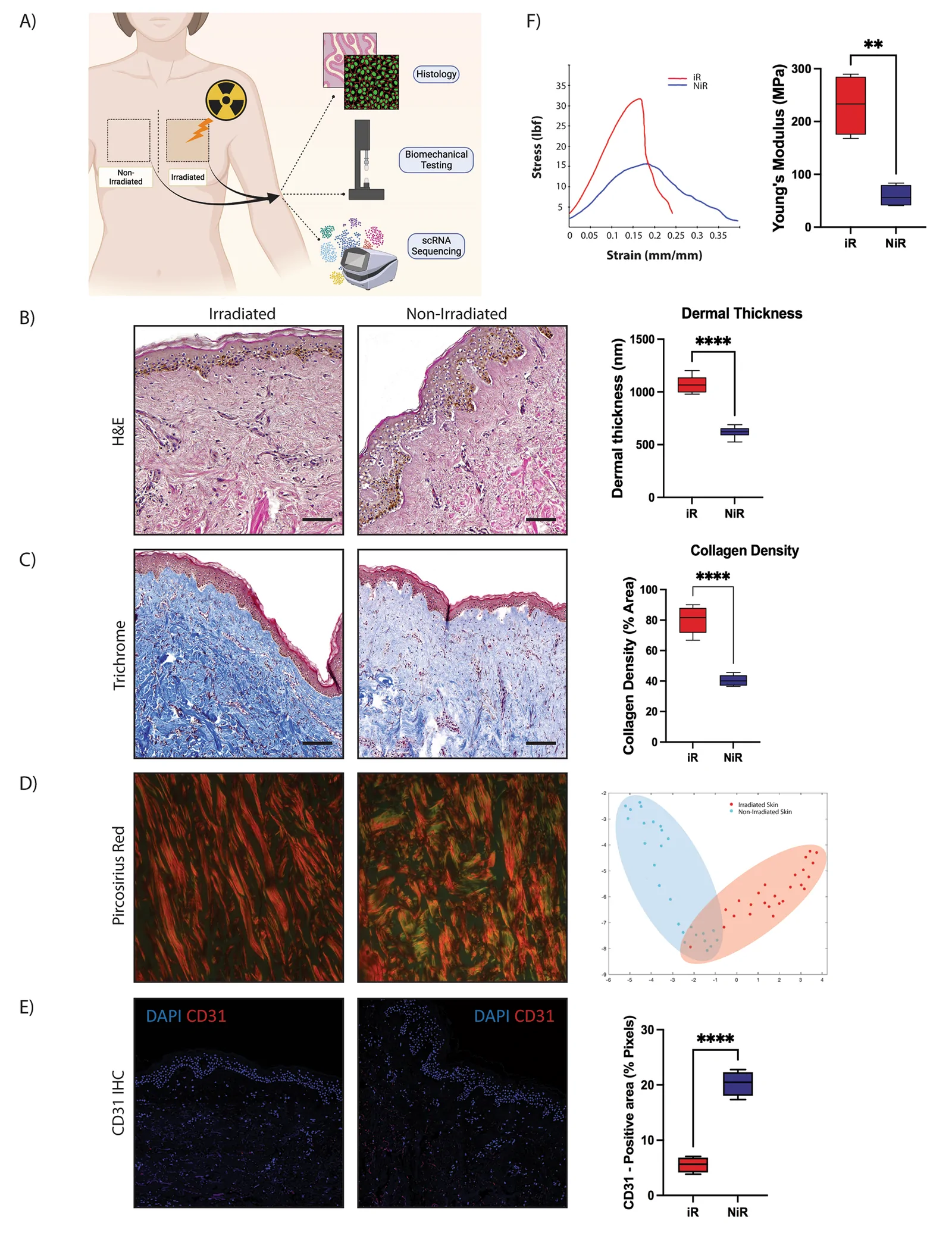
(**A**) Schematic of histological, biomechanical, and transcriptional analyses of irradiated and non-irradiated skin. (**B**) Representative 10x H&E images and quantification of dermal thickness in irradiated (left image) and non-irradiated (right image) skin. Dermal thickness was significantly increased in irradiated skin (red bar; *n* = 30/group; ****p < 0.0001). (**C**) Representative 10x trichrome images and quantification of collagen density in irradiated (left image) and non-irradiated (right image) skin. Collagen density was significantly increased in irradiated skin (red bar; *n* = 30/group; ****p < 0.0001). (**D**) Representative 40x Picrosirius red stained images and quantification of 294 extracellular matrix features with UMAP visualization (far right) for irradiated (red) and non-irradiated (blue) skin. Note irradiated skin ECM features clustered apart from non-irradiated skin. (**E**) Representative 20x CD31 immunofluorescent images and quantification. Irradiated skin (left image) had less CD31 staining (red) than non-irradiated skin (right image) (*n* = 15/group; ****p < 0.0001). (**F**) Representative stress-strain curves (left) of irradiated (iR, red line) and non-irradiated (NiR, blue line) skin. Irradiated skin (red bar) had higher young’s modulus (right chart) compared with non-irradiated skin (blue bar) (*n* = 6/group; **p < 0.01). Abbreviations: H&E, hematoxylin and eosin; iR, irradiated; NiR, non-irradiated; UMAP, uniform manifold approximation and projection. Scale bar = 200 μm

Skin specimens were stained with H&E, Masson’s trichrome, and Picrosirius Red (Fig. 1A). Inspection of H&E images showed that irradiated skin exhibited epidermal thinning in conjunction with significant dermal thickening (****p < 0.0001) (Fig. 1B). Collagen density of irradiated skin was also greater than that of non-irradiated skin, as shown by Masson’s Trichrome (****p < 0.0001) (Fig. 1C). With fibrosis seen in irradiated skin, the ECM demonstrated linearly organized, thick collagen fibers in contrast to the native basket-weave appearance observed in normal skin, as visualized with Picrosirius Red staining (Fig. 1D). Computational analysis of Picrosirius Red stained sections visualized on UMAP plot revealed that irradiated skin ECM features clustered apart from non-irradiated skin. Skin vascularity was evaluated by staining for CD31, and as expected, non-irradiated skin had greater CD31 staining than irradiated skin (****p < 0.0001) (Fig. 1E). Finally, paralleling histologic findings of increased dermal thickness and collagen density, mechanical testing revealed that irradiated skin exhibited a higher young’s modulus compared with non-irradiated skin (**p < 0.01) (Fig. 1F).

### Single-cell RNA sequencing of irradiated and non-irradiated skin

**Fig. 2:**
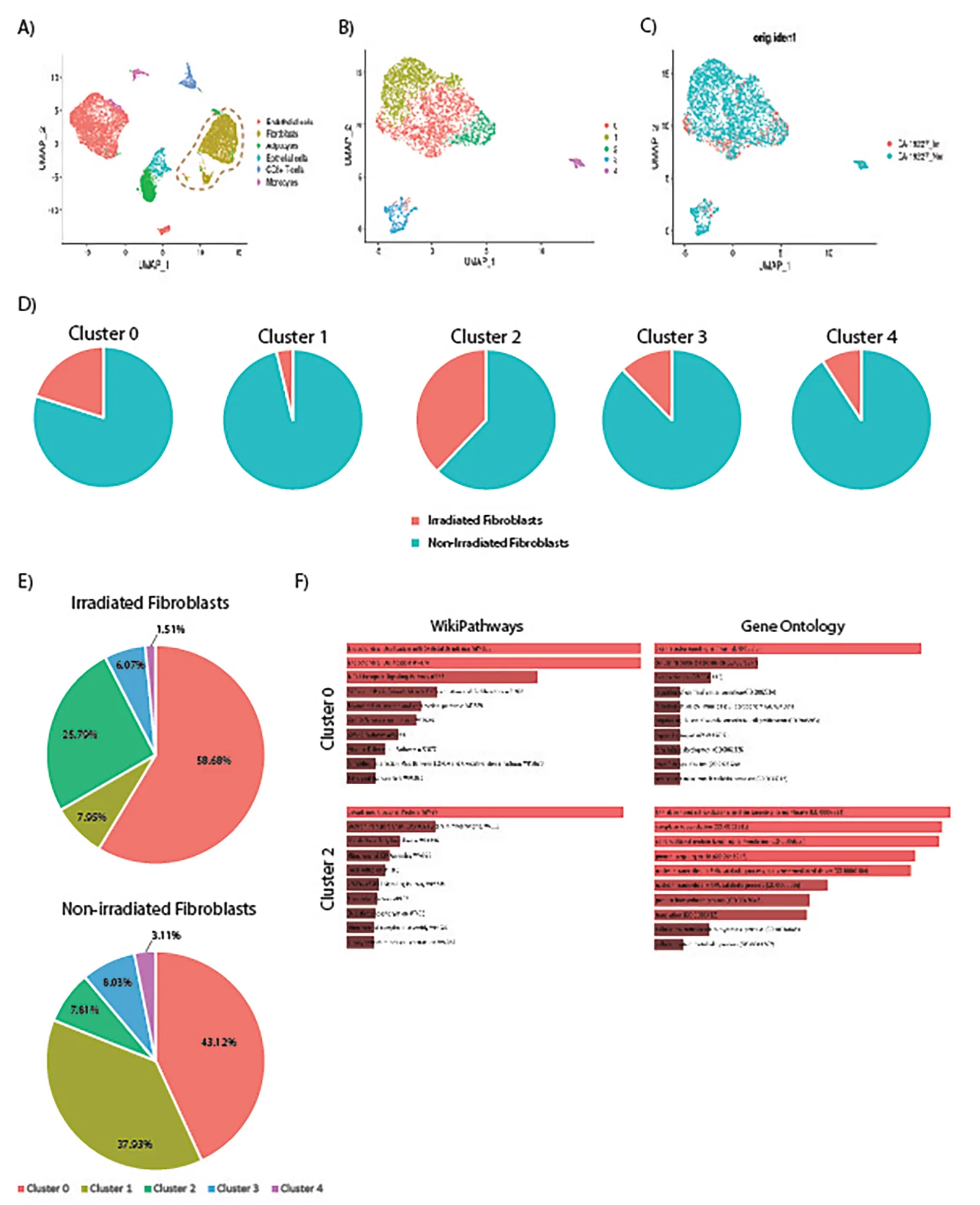
(**A**) Single cell transcriptional analysis of irradiated and non-irradiated skin with UMAP of identified cell types. Fibroblasts (dark yellow) shown within dotted line. (**B**) UMAP of five transcriptionally distinct fibroblast/stromal lineage clusters (Cluster 0–4). (**C**) UMAP plot to show the distribution of clusters in irradiated (red) and non-irradiated (blue) skin. (**D**) Pie charts to compare the distribution of irradiated (red) and non-irradiated (blue) cells within each cluster. (**E**) Pie charts comparing composition of irradiated (left) and non-irradiated (right) skin fibroblasts from each cluster. Note increased percentage of Cluster 0 (red) and Cluster 2 (green) cells among irradiated skin fibroblasts. (**F**) Pathways identified by enrichment analysis for Cluster 0 (top) and Cluster 2 (bottom). Abbreviations: UMAP, uniform manifold approximation and projection

Single-cell RNA sequencing of irradiated and non-irradiated skin samples revealed six distinct cell types identified using the automated annotation tool SingleR [19]. These included endothelial cells, fibroblasts, adipocytes, epithelial cells, CD8 T cells, and monocytes (Fig. 2A). To better evaluate heterogeneity among fibroblasts, these cells were isolated *in silico*, re-normalized independently of other cell types, and re-clustered. This identified five transcriptionally distinct fibroblast/stromal lineage subpopulations (Clusters 0–4) (Fig. 2B) based on differentially expressed genes for each cluster (Supplemental Table 1). Furthermore, distinct distribution of cell clusters in irradiated and non-irradiated skin was appreciated (Fig. 2C-D). Of note, increased prevalence of fibroblast cluster 2, and to a lesser extent cluster 0, was appreciated in irradiated skin (Fig. 2D-E). Enrichment analysis for cells in cluster 0 revealed biological processes related to lymphocyte signaling and function (Fig. 2F). Cluster 1 showed high expression levels of chemokine ligand 2 and suppressor of cytokine signaling 3, suggesting a role in regulating inflammation (Supplemental Table 1). This cluster also expressed high levels of CD44 and A Disintegrin and Metalloprotease family of proteins. Enrichment analysis showed pathways related to immunomodulatory cytokine mediated signaling (Supplemental Fig. 1).

Cluster 2 fibroblasts were found to be enriched for genes related to myocontractile functions such as myosin heavy chain 11, myosin light chain 9, smooth muscle actin alpha 2, and transgelin (Supplemental Table 1). Additionally, cluster 2 exhibited notable expression of both caveolin-1 (Cav1) and caveolin-2 (Cav2) (Supplemental Table 1). These genes belong to a family whose members encode the major proteins components of caveolae [20], which have been linked to vesicular trafficking, lipid metabolism, and regulation of mechanical signaling and ECM component production [21]. Pathways terms such as focal adhesion and protein targeting to membrane were identified with enrichment analysis (Fig. 2F).

Cells in clusters 3 and 4 were both more prevalent in non-irradiated skin samples (Fig. 2C-E). Cluster 3 displayed high expression levels of collagen type I alpha 1, collagen type III alpha 1, collagen type VI alpha 1, and the ECM proteins genes decorin, fibulin 1 and 2, and fibronectin suggesting a role in matrix deposition and remodeling. Consistent with our enrichment analysis, gene ontology (GO) terms for cluster 3 included extracellular matrix organization and positive regulation of cell proliferation (Supplemental Fig. 1). Cluster 4 cells were notable for their increased expression of genes including neurexin-1, cadherin 19, proteolipid protein 1, and sodium channel protein type 7. Enrichment analysis identified multiple nerve related terms such as nervous system development, axogenesis, and myelination (Supplemental Fig. 1), paralleling data supporting a role for innervation in limiting development of fibrosis [22]. 

### Radiation induces overexpression of Cav1 in fibroblasts

**Fig. 3:**
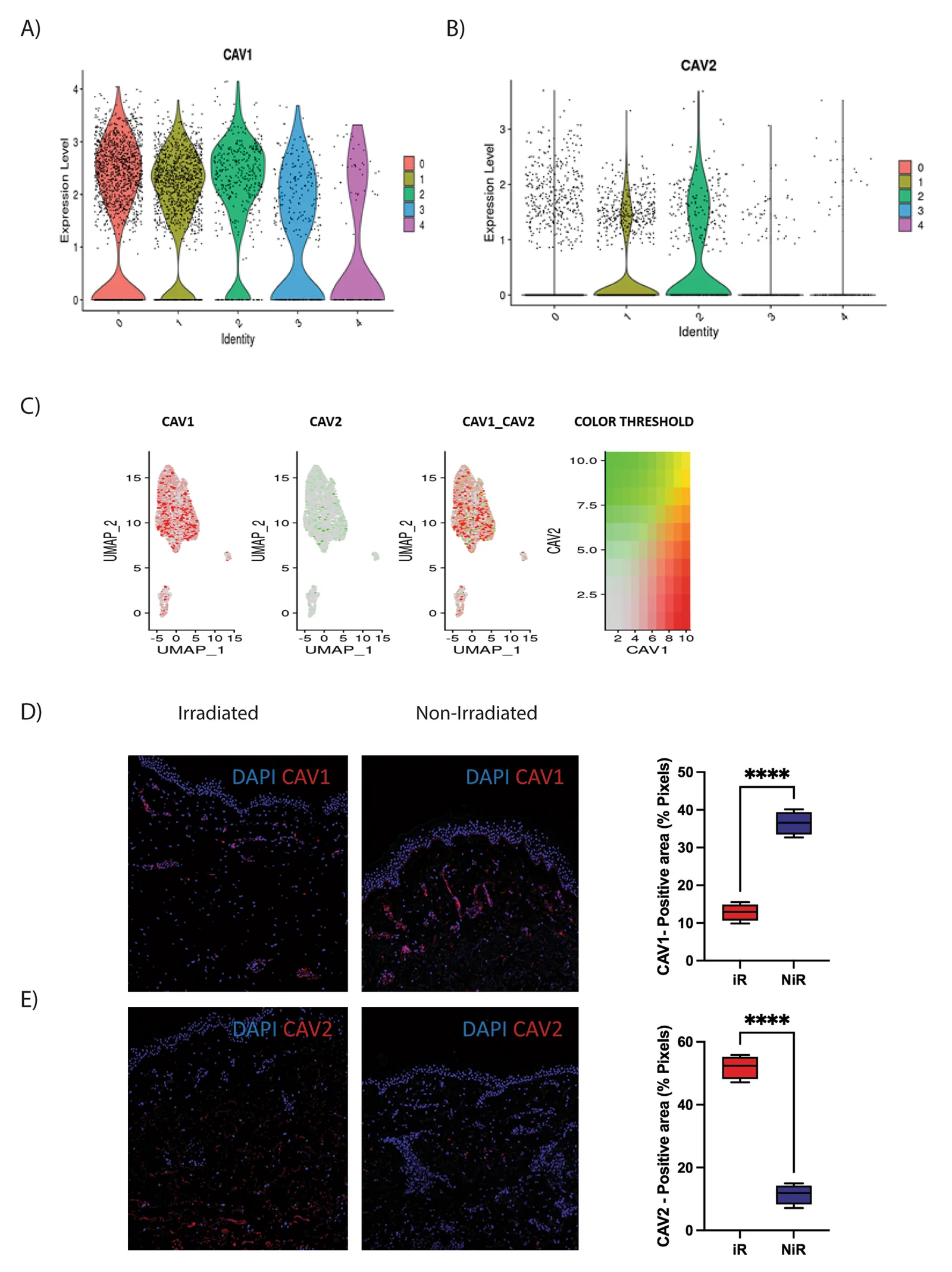
**A**) Violin plot showing expression of Cav1 across each fibroblast cluster. Note Cav1 expression was observed in all fibroblast clusters. **B**) Violin plot showing expression of Cav2 in each fibroblast cluster. Cav2 expression was primarily noted in Cluster 2 (green). **C**) Feature plots showing expression of Cav1 (red, left plot) and Cav2 (green, middle plot) for across all fibroblasts and where overlap expression was appreciated (right plot). **D**) Representative immunohistochemical staining images at 20x of Cav1 (red) with DAPI counterstain (blue) in irradiated (left image) and non-irradiated (right image) skin. Greater Cav1 staining was appreciated in non-irradiated skin (blue bar; n = 15/group; ****p < 0.0001). **E**) Representative immunohistochemical staining images at 20x for Cav2 (red) with DAPI counterstain (blue) in irradiated (left image) and non-irradiated (right image) skin. Staining for Cav2 was greater appreciated in irradiated skin (red bar; n = 15/group; ****p < 0.0001). Abbreviations: Cav1, caveolin 1; Cav2, caveolin 2; DAPI, 4’,6-diamidino-2-phenylindole; iR, irradiated; NiR, non-irradiated

Given that Cav1 and Cav2 are integral membrane proteins predominantly expressed in cells of the stromal components such as endothelial cells, adipocytes, and fibroblasts, we sought to gain more insight into the relevance of caveolin expression in fibroblasts following irradiation. First, we compared the expression level of Cav1 and Cav2 in each fibroblast cluster. Interestingly, Cav1 was expressed across all fibroblast clusters (0–4) (Fig. 3A), whereas Cav2 was predominantly expressed in cluster 2 (Fig. 3B). We then examined co-expression of both Cav1 and Cav2 across all fibroblast clusters, and consistent with the finding above, Cav1 expression was more readily appreciated in all clusters except cluster 2 where both Cav1 and Cav2 could be detected (Fig. 3C). We further assessed the expression of Cav1 and Cav2 in irradiated human skin by performing immunohistochemistry. While increased staining for Cav1 was found in non-irradiated skin (****p < 0.0001) (Fig. 3D), Cav2 was more readily appreciated in irradiated skin (****p < 0.0001) (Fig. 3E). Collectively, these results suggest that chronic histological and mechanical changes to skin induced by ionizing radiation are accompanied by a shift in the distribution of fibroblast subpopulations, increasing the prevalence of cells primed for mechanical signaling and with enhanced Cav2 expression.

## Discussion

Radiation-induced skin fibrosis is an important late effect of radiation therapy. Millions of oncologic patients worldwide are affected annually, and the resulting damaging effects of RIF can significantly impact reconstructive outcomes [11, 23, 24]. For breast cancer alone, there are over 1 million new diagnoses each year across the entire world, and with over 50% of patients receiving radiation therapy at some time during treatment of their disease, this equates to over 500,000 patients annually [25]. With breast reconstruction, prior radiation therapy with skin fibrosis may lead to tighter soft tissue envelope and increased rates of infection, implant exposure, and reconstructive failure [26, 27]. From the perspective of RIF pathogenesis, fibroblasts may be significantly involved in this process [28], and as fibroblasts are increasingly understood to be heterogeneous and highly plastic, understanding how radiation may impact their subpopulation dynamics may yield insights into biomolecular mechanisms driving development of chronic skin fibrosis and novel therapeutic avenues [29, 30]. 

Comparing irradiated and non-irradiated human skin, histologic changes induced by ionizing radiation, notably epidermal atrophy, dermal thickening, and decreased vascularization were readily appreciated. In addition, there was evidence of thick disorganized collagen deposition within the ECM of irradiated skin, as demonstrated by quantitative analyses using our novel unsupervised machine learning algorithm [31]. Not surprisingly, these histologic features were associated with stiffness that was higher in irradiated skin, a result that may be attributable to increased collagen density following radiation therapy.

Recent advances in single-cell transcriptomics have provided a high-resolution view of gene expression that can be applied to various organs, such as the skin, in order to delineate different cell types and states [32]. However, the application of single-cell transcriptional sequencing to explore radiation-induced skin fibrosis has remained lacking. On the basis of differential gene expression pattern, we identified six predominant cell types, and focusing on fibroblasts/stromal lineage cells, five distinct transcriptional clusters in irradiated and non-irradiated skin. Notably, cluster 2 was found to have the highest prevalence in fibrotic, irradiated skin compared to non-irradiated skin. Unique to this cluster was the overexpression of various cytoskeletal and mechanical signaling related genes including myosin heavy chain 11 and smooth muscle actin alpha 2. Enrichment analysis also identified terms such as focal adhesion pathway which supports a pro-fibrotic nature for this cluster given strong evidence in the literature for mechanotransduction driving adoption of scar forming characteristics within fibroblasts [8, 33, 34]. Cluster 0 prevalence was also noted to be increased in irradiated skin, and with a potential role in regulating inflammation, this subpopulation may mirror immunomodulatory cancer-associated fibroblasts which play in a role in formation of tumor desmoplasia [35]. 

While all fibroblast clusters were noted to express Cav1, cluster 2 fibroblasts were also found to interestingly express Cav2. Caveolae are composed of caveolins, cavins, and several other accessory proteins [36], and the caveolin proteins – Caveolin-1, Caveolin-2, and Caveolin-3 – serve as the main structural components of caveolae [37]. Cav1 is the best described member of the caveolin protein family, and Cav1 may influence ECM remodeling by promoting actin polymerization, stress fiber formation, and directional migration of fibroblasts [21]. In addition to participating in ECM remodeling, Cav1 may also regulate mechanical signaling through interactions with integrin-dependent signaling and focal adhesion assembly [21, 38]. Tissue remodeling and repair is dependent on modulating signaling between fibroblasts and the local environment, and Cav1 may play a role in balancing various exogenous stimuli on fibroblast behavior [39]. Importantly, studies have shown Cav1 to limit fibrosis in various organs, and this may in part be driven through regulation of transforming growth factor beta endocytosis and extracellular signal-regulated kinase/c-Jun N-terminal kinase activation [40, 41]. In the absence of Cav1, increased cardiac fibrosis and pulmonary fibrosis has been reported in animal models [42, 43]. And in radiation-induced lung injury, Cav1 deficiency was reported to promote fibroblast proliferation and collagen deposition [44, 45]. 

Whereas Cav1 is capable of forming homooligomers within caveolae, Cav2 forms monomers and dimers which localize to the Golgi apparatus, targeting this protein for degradation in the absence of Cav1 [46–50]. However, when Cav1 and Cav2 are co-expressed, Cav2 becomes stabilized and can redistribute to the caveolae membranes [51]. Specific roles for Cav2 in various cellular processes remain less well understood when compared to Cav1, but in mouse lung studies, Cav1 and Cav2 have been shown to have distinct function, with the balance between the two regulating the extent of bleomycin-induced fibrosis [52]. Emerging data thus suggest that Cav1 and Cav2 may act antagonistically to modulate inflammatory and fibrotic cellular responses [52]. In light of these findings, expression of Cav2 by cluster 2 fibroblasts in our study may underscore the pro-fibrotic behavior of these cells which demonstrated increased prevalence in irradiated tissue.

A key limitation to this study is the retrospective nature of irradiated skin sample acquisition and the variability in time point following radiation therapy when each paired specimen was obtained. While all samples demonstrated histologic features of chronic, radiation-induced fibrosis, post-radiation soft tissue injury is a dynamic process with continual evolution of cytokine signaling and cellular turnover. Ongoing changes, even in the late, chronic stage when irradiated skin was obtained, may impair the ability to fully characterize behavior of specific fibroblast subpopulations in our study. Additionally, all samples were acquired from female patients undergoing breast reconstruction, and this may also limit translatability of our findings to all other patients with RIF.

## Conclusions

Single-cell transcriptional sequencing of paired irradiated and non-irradiated human skin revealed specific changes to fibroblast subpopulations which may contribute to development of fibrosis in response to radiation therapy. This study thus provides a framework to further expand on the current knowledge in the field of RIF. By identifying potential molecular targets such as caveolins, more precise strategies for RIF prevention and treatment may be developed.

## Electronic supplementary material

Below is the link to the electronic supplementary material.

Supplementary Material 1: Supplemental Fig. 1. Enrichment analysis for fibroblast cluster 1 (top), cluster 3 (middle), and cluster 4 (bottom) with pathways shown from WikiPathways functional annotation of biological pathways (left) and Gene Ontology (GO) terms of biological processes for genes that are upregulated (right).

Supplementary Material 2: Supplemental Table 1. Top differentially expressed genes for each cluster

## Data Availability

Datasets generated and used and/or analyzed during the current study are available from the corresponding author on reasonable request. Gene expression datasets generated during this study are publicly available and can be found through Gene Expression Omnibus (GEO) web portal. The GEO accession number for this study’s datasets is GSE341487.

## Abbreviations

Cav1: caveolin 1
Cav2: caveolin 2
DEGs: differentially expressed genes
ECM: extracellular matrix
H&E: hematoxylin & eosin
Picro: picrosirius red
RIF: radiation induced fibrosis
scRNA: seq
TC: Masson’s Trichrome
UMAP: Uniform Manifold Approximation and Projection
