# Supplementary figures and images for: Single-cell transcriptional analysis of irradiated skin reveals changes in fibroblast subpopulations and variability in caveolin expression
Source: Radiat Oncol. 2024 Jun 26;19:82. doi: 10.1186/s13014-024-02472-z (PMC11200992; doi:10.1186/s13014-024-02472-z)

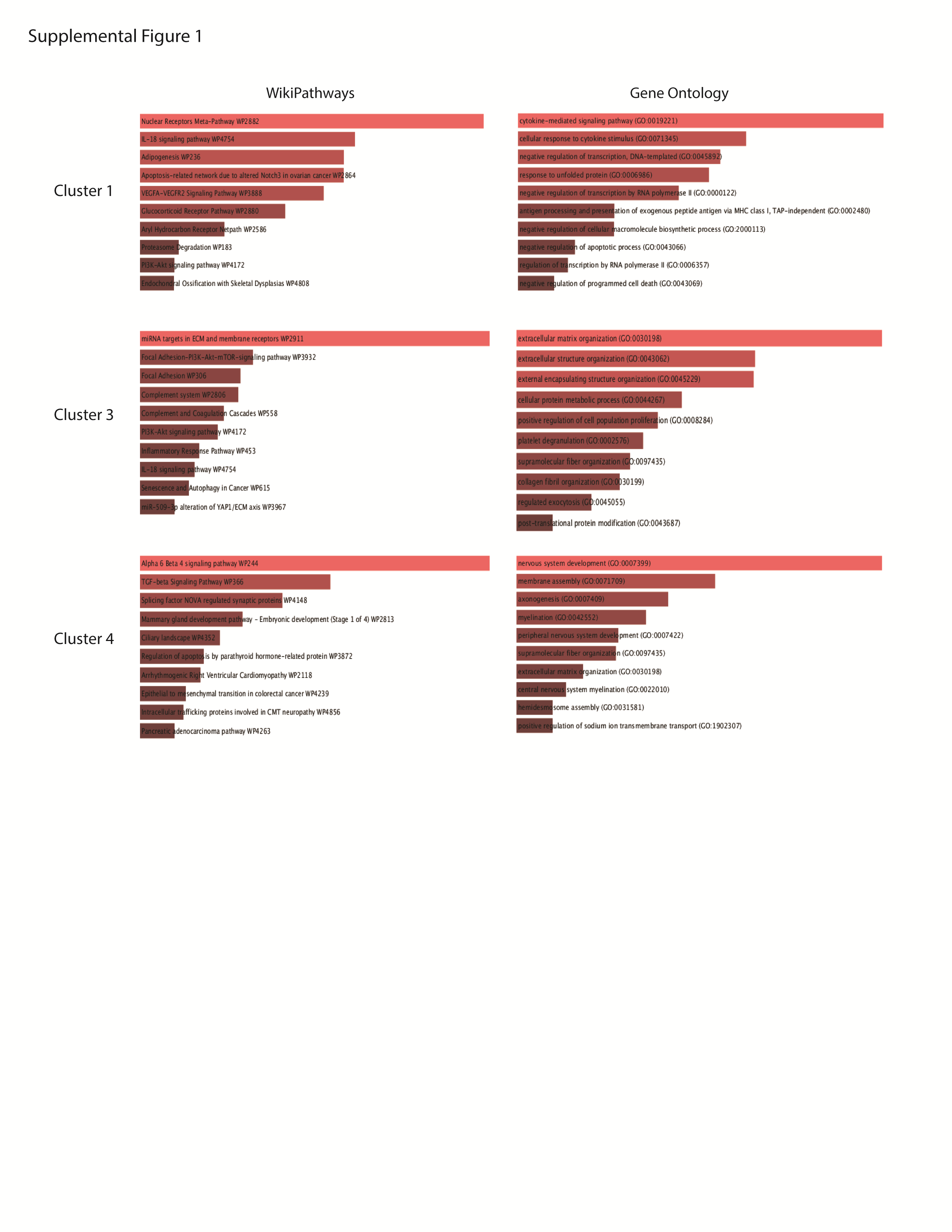

Supplement: Supplementary file 1 — Supplementary Material 1: Supplemental Fig. 1. Enrichment analysis for fibroblast cluster 1 (top), cluster 3 (middle), and cluster 4 (bottom) with pathways shown from WikiPathways functional annotation of biological pathways (left) and Gene Ontology (GO) terms of biological processes for genes that are upregulated (right). [file 13014_2024_2472_MOESM1_ESM.png]
