# Supplementary material for: Single-cell transcriptional analysis of irradiated skin reveals changes in fibroblast subpopulations and variability in caveolin expression
Source: Radiat Oncol. 2024 Jun 26;19:82. doi: 10.1186/s13014-024-02472-z (PMC11200992; doi:10.1186/s13014-024-02472-z)
Supplement: Supplementary file 2 — Supplementary Material 2: Supplemental Table 1. Top differentially expressed genes for each cluster [file 13014_2024_2472_MOESM2_ESM.docx]

**Supplemental Table 1**

***CLUSTER 0***

| **Symbol** | **Gene Name** | **Fold Change** | ***p*-Value** |
| --- | --- | --- | --- |
| *MTRNR2L12* | MT-RNR2 like protein 12 | 1.53921642 | 9.48E-43 |
| *FABP4* | fatty acid binding protein 4 | 6.35133224 | 6.82E-27 |
| *MTRNR2L8* | MT-RNR2 Like Protein 8 | 1.70471698 | 1.62E-23 |
| *PLCG2* | phospholipase C gamma 2 | 1.95418961 | 1.64E-20 |
| *RGS5* | regulator of G protein signaling 5 | 1.36865621 | 1.24E-19 |
| *STEAP4* | six-transmembrane epithelial antigen of prostate 4 | 2.05162554 | 3.57E-18 |
| *MTATP8* | mitochondrially encoded ATP synthase membrane subunit 8 | 1.58103852 | 2.21E-05 |
| *DCD* | dermcidin | 1.56955297 | 4.28E-05 |
| *SCGB2A2* | secretoglobin family 2A member 2 | 1.54959407 | 0.00088675 |
| *HIGD1B* | HIG1 hypoxia inducible domain family member 18 | 1.7969627 | 0.00390479 |
| *FOXK2* | forkhead box K2 | 1.19583744 | 0.00414895 |
| *SBF2* | SET binding factor 2 | 1.23377178 | 0.00445739 |
| *FMR1* | fragile X messenger ribonucleoprotein 1 | 1.21342 | 0.00468588 |
| *PELI1* | pellino E3 ubiquitin protein ligase 1 | 1.24160896 | 0.00491802 |
| *PLAU* | plasminogen activator, urokinase | 2.66456663 | 0.00516306 |

***CLUSTER 1***

| **Symbol** | **Gene Name** | **Fold Change** | ***p*-Value** |
| --- | --- | --- | --- |
| *RGS16* | regulator of G protein signaling 16 | 3.03262697 | 4.09E-229 |
| *MT1A* | metallothionein 1A | 2.99584939 | 4.36E-208 |
| *SERTAD1* | SERTA domain containing protein 1 | 3.37642693 | 5.21E-202 |
| *CCL2* | c-c motif chemokine ligand 2 | 3.31082321 | 6.45E-201 |
| *CDKN1A* | cyclin dependent kinase inhibitor 1A | 2.74768576 | 2.93E-188 |
| *DDIT4* | DNA damage inducible transcript 4 | 3.03091397 | 7.77E-185 |
| *CYP26B1* | cytochrome p450 family 26 subfamily B member protein 1 | 3.28991741 | 2.00E-180 |
| *ID4* | inhibitor of DNA binding 4 | 2.92031095 | 3.76E-175 |
| *SOCS3* | suppressor of cytokine signaling 3 | 2.56442471 | 8.09E-174 |
| *HAS2* | hyaluronan synthase 2 | 5.19850588 | 6.48E-173 |
| *IRF1* | interferon regulatory factor 1 | 2.36002883 | 9.28E-165 |
| *ADAMTS4* | A disintegrin and metalloprotease with thrombospondin motif 4 | 2.1449739 | 5.50E-111 |
| *IL6* | interleukin 6 | 2.60797031 | 1.88E-74 |
| *CCL8* | c-c motif chemokine ligand 8 | 4.45466576 | 4.63E-64 |
| *CD44 / PGP1* | phagocytic glycoprotein 1 | 1.46142501 | 7.20E-45 |

***CLUSTER 2***

| **Symbol** | **Gene Name** | **Fold Change** | ***p*-Value** |
| --- | --- | --- | --- |
| *RERGL* | ras-related and estrogen-regulated growth inhibitor-like protein | 70.0747762 | <1.00E-300 |
| *MYH11* | myosin heavy chain 11 | 21.5499776 | <1.00E-300 |
| *PLN* | phaspholamban | 54.5055579 | 1.48E-270 |
| *NET1* | neuroepithelial cell transforming protein 1 | 9.92363073 | 2.66E-210 |
| *DSTN* | destrin, actin depolymerizing factor | 5.35313169 | 1.90E-161 |
| *SORBS2* | sorbin and SH3 domain containing protein 2 | 9.19165537 | 4.69E-161 |
| *ACTA2* | smooth muscle actin alpha 2 | 3.77305126 | 3.87E-113 |
| *TPM2* | tropomyosin 2 | 3.67338875 | 1.86E-104 |
| *NDUFA4* | NADH-ubiquinone oxidoreductase MLRQ subunit | 3.98286062 | 5.01E-102 |
| *SNCG* | synuclein gamma | 5.27756279 | 1.85E-79 |
| *TGLN* | transgelin | 2.12570606 | 2.09E-77 |
| *MYL9* | myosin light chain 9 | 2.1928003 | 3.69E-68 |
| *LTBP1* | latent transforming growth factor beta binding protein 1 | 6.24507571 | 2.55E-48 |
| *CAV2* | caveolin 2 | 1.86319374 | 8.98E-18 |
| *CAV1* | caveolin 1 | 1.28170417 | 2.72E-09 |

***CLUSTER 3***

| **Symbol** | **Gene Name** | **Fold Change** | ***p*-Value** |
| --- | --- | --- | --- |
| *CXCL14* | c-x-c motif chemokine ligand 14 | 223.138643 | <1.00E-300 |
| *APOD* | apolipoprotein D | 67.3492221 | <1.00E-300 |
| *PTGDS* | prostaglandin D2 synthase | 155.336869 | <1.00E-300 |
| *SFRP2* | secreted frizzled related protein 2 | 119.571826 | <1.00E-300 |
| *FBLN1* | fibulin 1 | 65.8952292 | <1.00E-300 |
| *VCAN* | versican | 44.5636875 | <1.00E-300 |
| *C3* | complement C3 | 234.895912 | <1.00E-300 |
| *MMP2* | matrix metallopeptidase 2 | 94.5170564 | <1.00E-300 |
| *CFD* | complement factor D | 40.2263999 | 7.17E-296 |
| *DCN* | decorin | 43.9684272 | 2.04E-291 |
| *FBLN2* | fibulin 2 | 21.9642404 | 1.00E-196 |
| *COL1A1* | collagen type 1 alpha 1 | 8.87714661 | 1.07E-90 |
| *COL3A1* | collagen type 3 alpha 1 | 6.10155504 | 3.98E-72 |
| *FN1* | fibronectin 1 | 6.09467588 | 2.43E-67 |
| *COL6A1* | collagen type 6, alpha 1 | 2.99065782 | 1.11E-54 |

***CLUSTER 4***

| **Symbol** | **Gene Name** | **Fold Change** | ***p*-Value** |
| --- | --- | --- | --- |
| *NRXN1* | neurexin 1 | 1207.89337 | <1.00E-300 |
| *CDH19* | cadherin 19 | 414.392227 | <1.00E-300 |
| *S100B* | S100 calcium binding protein B | 331.418734 | <1.00E-300 |
| *SCN7A* | sodium channel protein type 7, alpha subunit | 274.420074 | <1.00E-300 |
| *PLP1* | proteolipid protein 1 | 615.999596 | <1.00E-300 |
| *PCSK2* | proprotein convertase subtilisin/kexin type 2 | 601.314473 | 4.02E-306 |
| *MT3* | metallothionein 3 | 401.957403 | 1.32E-267 |
| *MPZ* | myelin protein zero | 467.642541 | 9.05E-215 |
| *SCN9A* | sodium channel protein type 9, alpha subunit | 169.448775 | 1.98E-108 |
| *COL9A3* | collagen type 9, alpha 3 | 148.994289 | 2.00E-89 |
| *ITGB4* | integrin subunit beta 4 | 40.1352247 | 4.60E-81 |
| *NCAM1* | neural cell adhesion molecule 1 | 96.9511047 | 3.55E-80 |
| *CLU* | clusterin | 16.0806298 | 5.97E-64 |
| *CRYAB1* | crystallin alpha B | 20.5012385 | 1.65E-60 |
| *NRXN3* | neurexin 3 | 192.907624 | 2.41E-60 |
